# Mucopolysaccharidoses: early diagnostic signs in infants and children

**DOI:** 10.1186/s13052-018-0550-5

**Published:** 2018-11-16

**Authors:** Cinzia Galimberti, Annalisa Madeo, Maja Di Rocco, Agata Fiumara

**Affiliations:** 1 Department of Pediatrics, Fondazione MBBM, San Gerardo Hospital, Monza, Italy; 2 Unit of Rare Diseases, Department of Pediatrics, Gaslini Institute, Genoa, Italy; 3 Regional Referral Centre for Metabolic Diseases, Pediatric Clinic, Department of Clinical and Experimental Medicine, University of Catania, AOU Policlinico VE, Via Santa Sofia 78, 95123 Catania, Italy

**Keywords:** Mucopolysaccharidosis, MPS, Early-onset MPS, Early MPS signs, Early MPS symptoms

## Abstract

Mucopolysaccharidoses (MPS) comprise a group of lysosomal disorders that are characterized by progressive, systemic clinical manifestations and a coarse phenotype. The different types, having clinical, biochemical, and genetic heterogeneity, share key clinical features in varying combinations, including joint and skeletal dysplasia, coarse facial features, corneal clouding, inguinal or abdominal hernias, recurrent upper respiratory tract infections, heart valve disease, carpal tunnel syndrome, and variable neurological involvement. In the severe forms, these features usually appear in the first months of life, but a correct diagnosis is often reached later when suggestive signs are manifest. All MPS types may have severe or attenuated presentations depending on the residual enzymatic activity of the patient. Based on data from the literature and from personal experience, here we underline the very early signs of the severe forms which should alert the paediatrician on their first appearance. A few early signs are typical of MPS (i.e. gibbus) while many are unspecific (hernias, upper airway infections, organomegaly, etc.), and finding the association of many unspecific signs might prompt the paediatrician to search for a common cause and to carefully look for other more specific signs (gibbus and other skeletal deformities, heart murmur). We stress the need to increase awareness of MPS among paediatricians and other specialists to shorten the still existing diagnostic delay. A timely diagnosis is mandatory for the commencement of treatment as soon as possible, when available, to possibly obtain better results.

## Background

Mucopolysaccharidoses (MPS) are a group of clinically heterogeneous diseases caused by deficiencies of the lysosomal enzymes required for the breakdown of glycosaminoglycans (GAGs).

MPS are characterized by progressive and systemic clinical manifestations. Despite their biochemical and genetic heterogeneity, different types share key clinical features in varying combinations, including joint and skeletal dysplasia with stiffness (except MPS IV where there is laxity) and pain, coarse facial features, corneal clouding, inguinal or abdominal hernias, recurrent upper respiratory tract infections, heart valve disease, carpal tunnel syndrome, and variable neurological involvement. These features usually appear in the first months of life for the severe forms and in early childhood for the more attenuated forms, but are often underestimated and generally taken into account only when clearly evident.

The rarity of these disorders and the variability in clinical presentation frequently leads to a diagnostic delay; this may range from months, for the severe forms, to years, for the attenuated ones (see Rigoldi et al. in this supplement [1]). Nevertheless, due to the fast progression and urgent need for intervention in the severe presentations, even months of delay in diagnosis may produce catastrophic results for the future health of the patient.

Our aim is to underline in this review the very early signs of the severe forms which should alert the paediatrician on their first appearance.

### What signs/symptoms can we expect in children with severe forms of MPS?

**Fig. 1:**
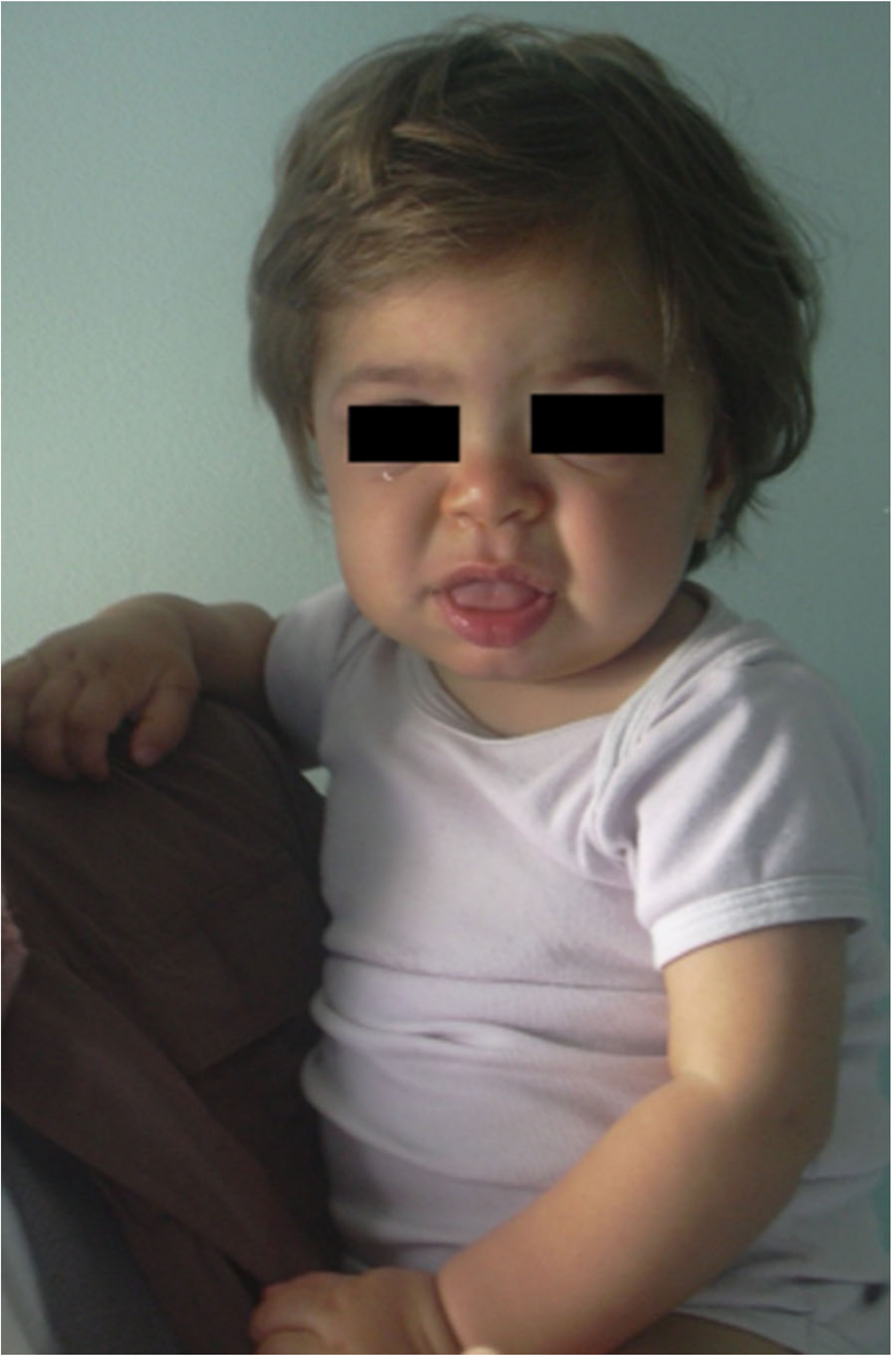
MPS IH in 1-year-old patient. Coarse facies: flat nasal bridge, macroglossia, frontal bossing

Symptoms and signs suggestive of different forms of MPS are reported in Table 1 according to age. In the first 6 months of life, inguinal hernias, abnormally frequent respiratory infections, otitis, and organomegaly can be seen in MPS patients, particularly in MPS I (Hurler syndrome) which has the most precocious severe onset. Moreover, from 6 to 12 months, these infants very often develop a gibbus (toraco-lumbar kyphosis), heart murmur, umbilical hernia, mild hypotonia, and growth delay [2]. They may also have the typical coarse facial appearance (Fig. 1). MPS II, VI, and VII may present with a similar early phenotype. MPS IV patients have an early skeletal phenotype with the appearance of hip dysplasia in the first months of life and pigeon chest (pectus carinatum) in the first 12–18 months. MPS II infants present with a similar involvement but with later onset. In the second year of life, MPS I Hurler syndrome infants develop cognitive delay while an MPS II patient might be identified only because of overgrowth, frequent airway infections, and multiple surgeries [3, 4]; the typical facial features appear only later. Some MPS II patients also develop hyperactivity between 18 and 24 months. In the third year of life, hyperactivity and cognitive delay are easily recognizable in MPS II and MPS III.

**Table 1:** Age of onset of the main signs and symptoms in different mucopolysaccharidosis (MPS) types

Sign/ symptom	Age of onset 0-24 months	Age of onset >2 yrs	MPS
Hydrops foetalis [10, 19]	Birth		I, IVA, VII
Diarrhea [6, 13]	First months		I, II, III
Hernias [11, 12]	Birth or first months		Almost all MPS
Upper respiratory hypersecretion/Infections [6, 13]	First months –1 Year		I, II, III, VI
Heart valve [8, 13, 18, 21]	< 1 year		I
		< 5 yrs	II, VI, VII
Coarse face [11, 15]	9-18 m		I,II, VI
		2-4 yrs	II, III
Corneal clouding [11]	< 15 m		I, VI
Liver/spleen enlargement [6, 13]	< 1 year		I,II,III, VI
Cognitive delay [2, 12]	< 18 months		I
		2-3 years	II,III
Macrocrania [1, 6, 21]	Birth – 1 year		I, II, III, IV, VI, VII
Skeletal dysplasia [2, 15]	< 1 year		II, IV
		2- 4 yrs	I, VI
Growth impairment [13]	12 – 18 m		I
		<3 yrs	IV, VI
Behavioural anomalies [1, 6]	< 2 yrs		I
		2-4 years	II, III
Sleep disturbances [6, 13]	First year		I,II,III, VI

In summary, skeletal manifestations are typical and sometimes precocious. Coarse facies and cognitive delay are not early prominent signs.

### What are the different presentations of the various types of MPS?

#### MPS with somatic and cognitive involvement

**Fig. 2:**
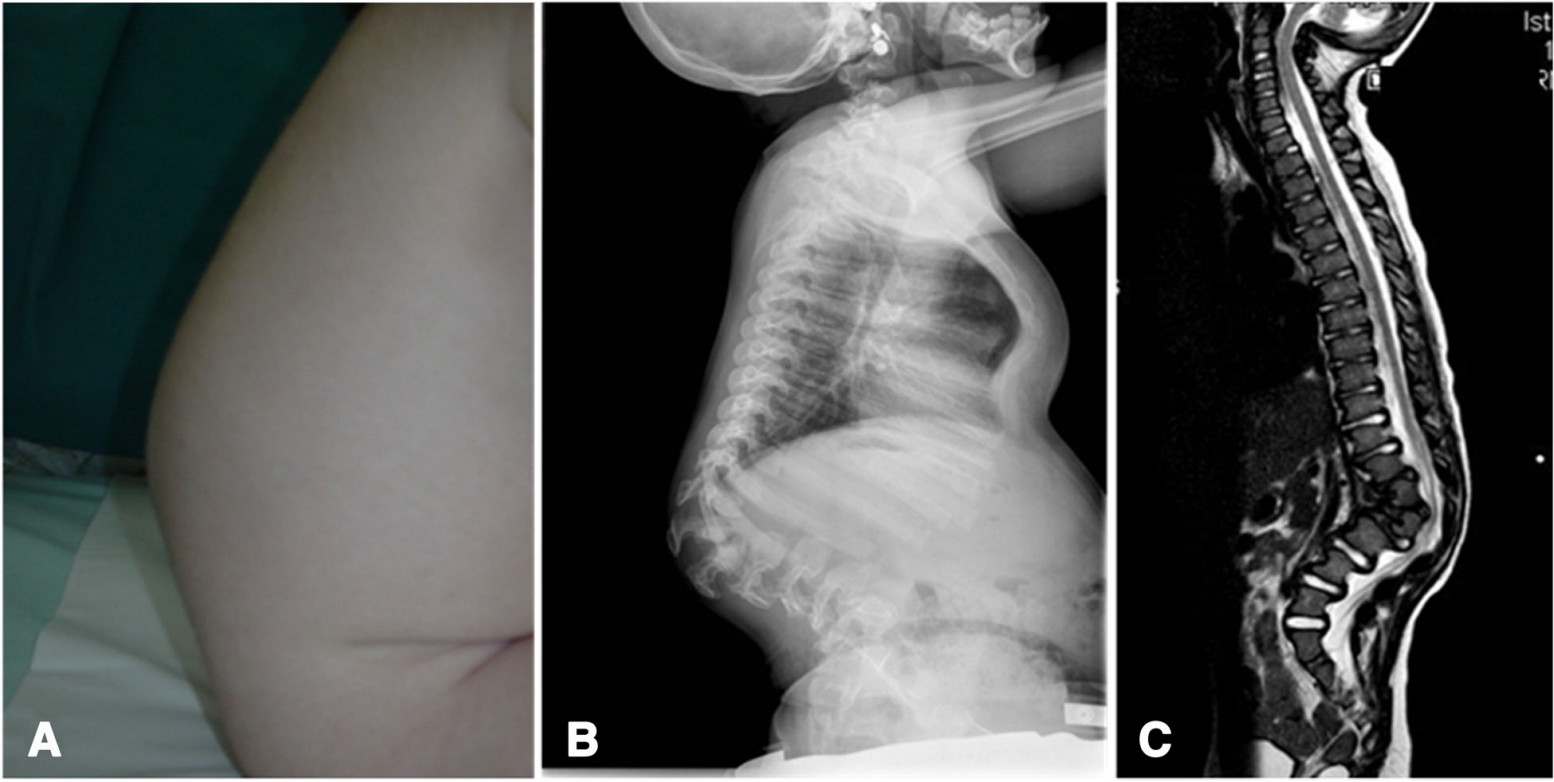
MPS IH. **a** Gibbus in a 9-month-old patient. **b** Spine x-ray of the spine in a 3-year-old patient. **c** T2-weighted magnetic resonance image of the spine showing significant kyphosis and vertebral canal stenosis

**Fig. 3:**
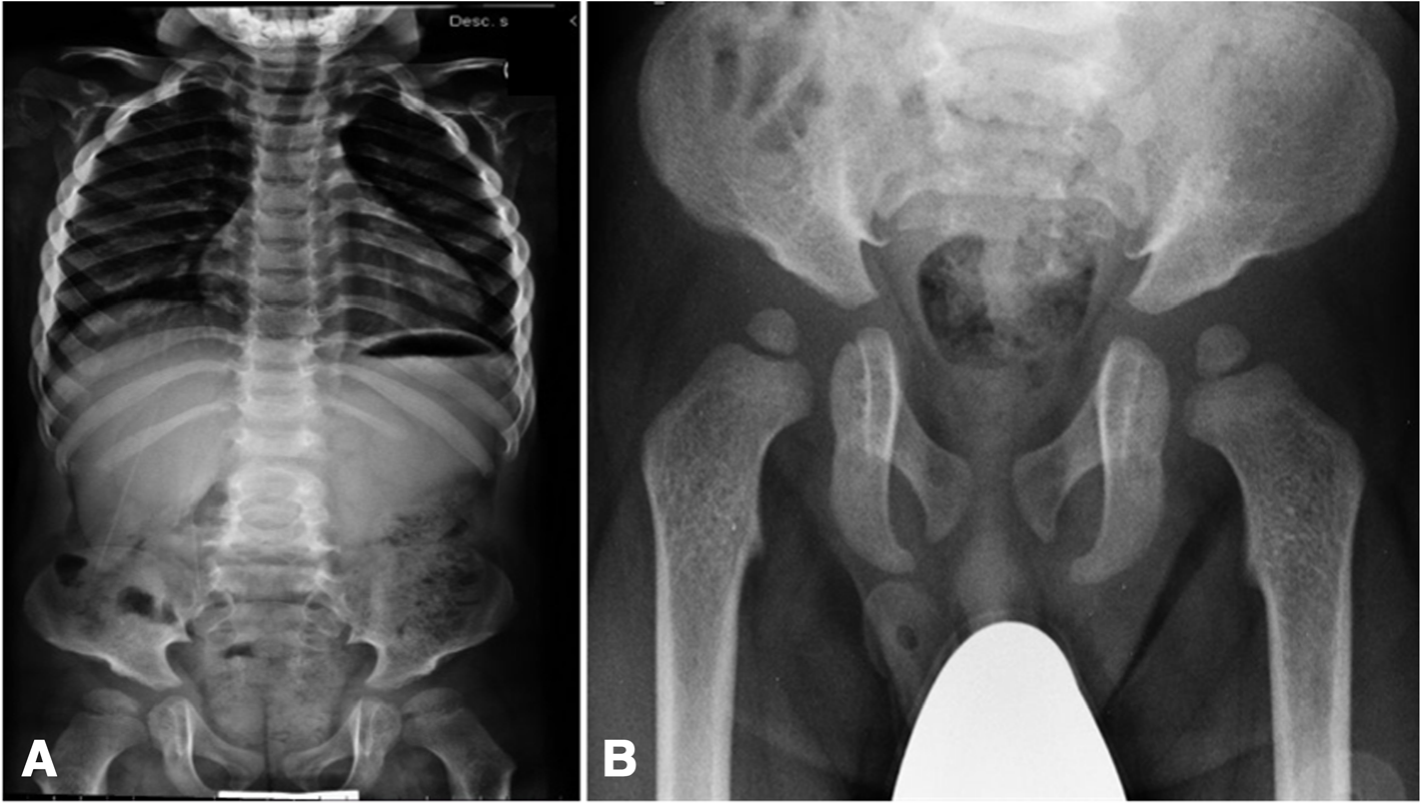
MPS IH. X-ray evidence of dysostosis multiplex. **a** Thickening of the ribs in a 3-year-old patient and **b** hip dysplasia in another patient aged 18 months

**Fig. 4:**
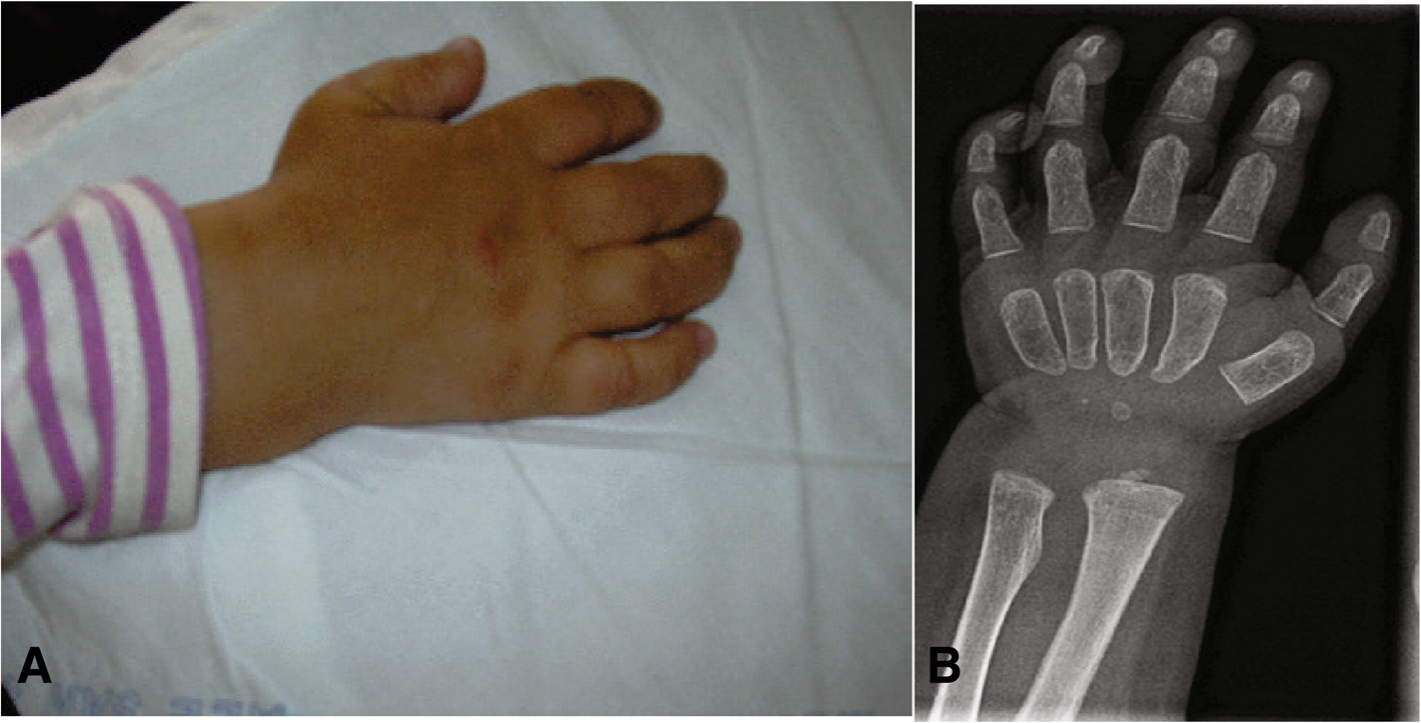
MPS IH: **a** Claw hand in an 18 month-old patient; **b** hand X-ray.

**Fig. 5:**
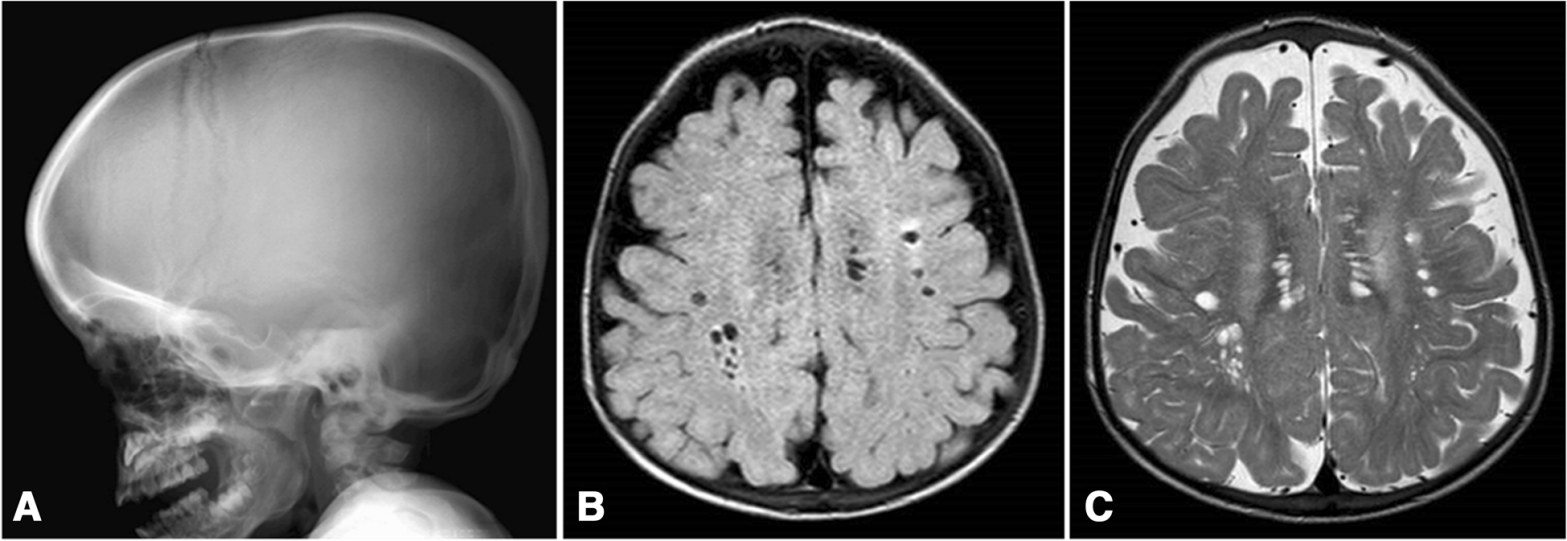
MPS IH. **a** Thickening of the cortical bone of the skull and abnormal “J-shaped” conformation of the sella turcica. Dilatation of the periventricular spaces in **b** FLAIR and **c** T-2 weighted magnetic resonance images of a patient aged 17 months

Mucopolysaccharidosis type I (MPS I; OMIM #252800) (Figs. 1, 2, 3, 4 and 5) is caused by a deficiency of the lysosomal hydrolase α-l-iduronidase, which leads to multisystem accumulation of two GAGs: dermatan sulphate (DS) and heparan sulphate (HS) [3]. Based on its severity, MPS I is traditionally divided into three clinical phenotypes; however, these forms represent a continuum of disease severity. Hurler syndrome, the most severe form, typically presents during the first year of life. Affected children rapidly develop significant cognitive impairment and somatic disease in multiple organ systems, leading to death within the first decade in the absence of treatment. The attenuated forms of MPS I, known as Hurler–Scheie syndrome and Scheie syndrome, respectively, are characterized by later onset of symptoms, longer life expectancy, and mild or no central nervous system (CNS) involvement [2]. Inheritance is autosomal recessive, and in at least 50% of cases phenotype prediction is possible on the basis of genotype while in the remaining 50% new mutations are detected [5]. Prevalence is approximately 1 in 100,000 live births [6].

**Fig. 6:**
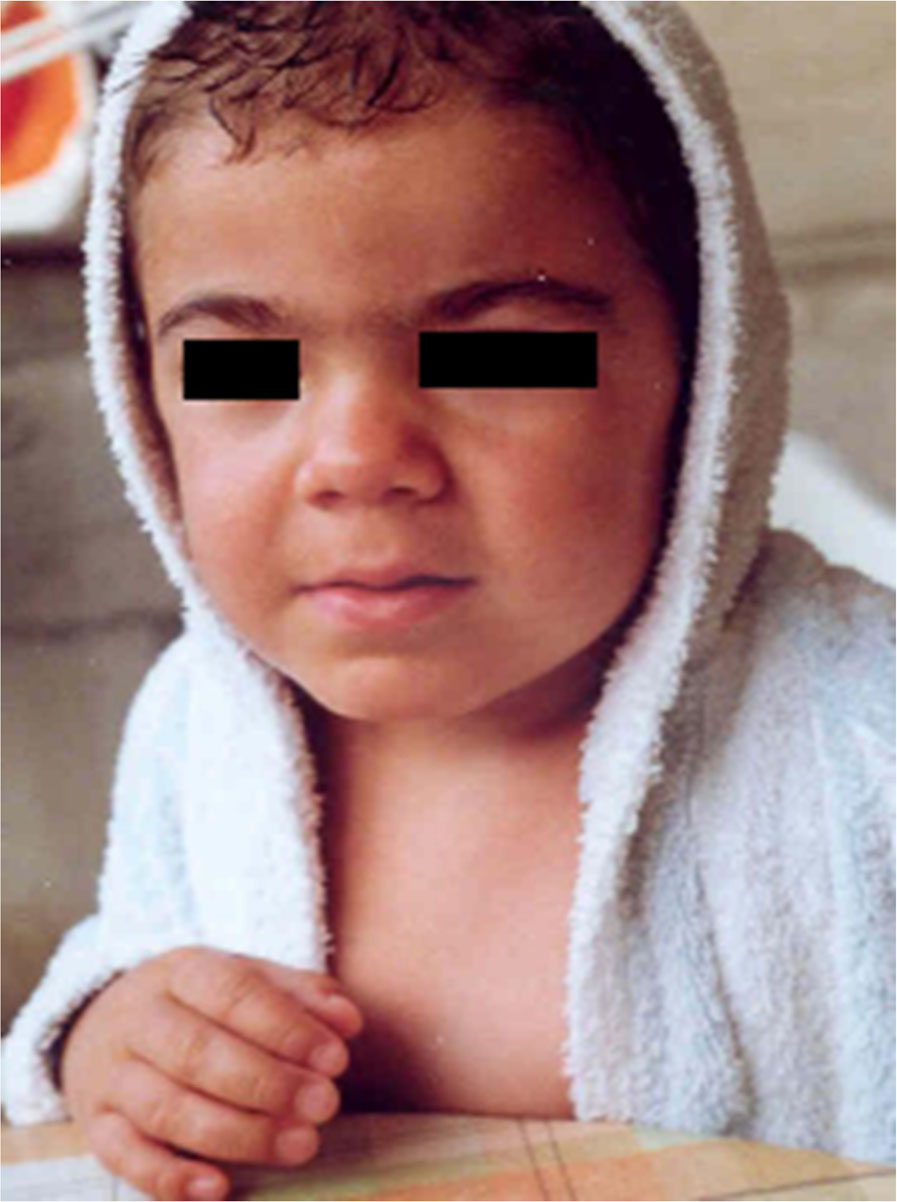
MPS II. A 3-year-old patient with only mild characteristic facial features

Mucopolysaccharidosis type II (MPS II; OMIM #309900) (Fig. 6), also known as Hunter syndrome, is the result of deficiency of the enzyme iduronate-2-sulfatase (I2S) with consequent GAG accumulation of DS and HS [7]. Prevalence is around 1 in 140,000–156,000 male live births [8]. Notably, this is the only X-linked MPS, and female carriers are usually asymptomatic; however, they can be exceptionally affected due to abnormalities of the X-chromosome, homozygosity, or skewed-X-inactivation [9]. The phenotypic expression also spans a wide spectrum of clinical severity. In severe cases, marked progressive neurologic involvement and somatic disease co-exist, and death usually occurs in the second decade of life. Other patients with minimal or no neurological dysfunction may present a severe somatic involvement while, at the opposite end of the spectrum, there are patients with only minimal somatic manifestations and normal intelligence who survive into adulthood [10]. Early signs and symptoms are shared by the severe forms of MPS I and II.

Sporadic cases of hydrops fetalis are described in MPS I [11], although, in general, these children appear normal at birth. The first signs of MPS I usually appear during the very first months of life, while those in MPS II usually occur a bit later [10]. Kiely et al. [2], in a retrospective study in 55 Hurler patients, observed that respiratory symptoms, feeding difficulties, inguinal hernia, and otitis media had appeared at a median age ≤ 6 months, and kyphosis, cardiac abnormalities, enlarged head circumference, hypotonia, organomegaly, corneal clouding, joint restriction, and umbilical hernia appeared between 6 and 12 months. Umbilical hernia is one of the earliest presenting features in MPS I and II, and inguinal hernias are reported in approximately 60% of patients [12, 13].

Another common initial clue for MPS I and II can be upper airway recurrent infections with increased secretions. Frequent otitis, chronic recurrent rhinitis, and persistent nasal discharge without obvious infection are not specific, but may help in strengthening the diagnostic suspicion if associated with more specific signs. Storage of GAG within the oro-pharynx leads to enlargement of the tonsils and adenoids, and contributes to upper airway complications. Another frequent finding is noisy breathing, particularly at night, associated in the later stages with obstructive sleep apnoea. As a consequence of frequent middle-ear infections and dysostosis of the ossicles of the middle ear, hearing loss is frequent, with both conductive and sensorineural deficits [8, 14]. Tracheobronchomalacia is commonly observed and can lead to acute airway obstruction or collapse, with this being one of the main causes of death in subsequent years [13].

Adenoidectomy, tonsillectomy, and tympanostomy, together with hernia repair, are the more frequent and early surgical interventions for MPS I and II patients, often performed before knowing the diagnosis (in more than 50% of cases) [15].

Recurrent diarrhoea is quite common in early stages of MPS I, and can be also present in MPS II patients with neurologic involvement, while it is uncommon in the mildly affected patients [2, 9].

Gibbus deformity (dorso-lumbar kyphosis) (Fig. 2) becomes clinically apparent at a median age of 8.6 months to 1 year in MPS I [1, 12], representing a quite specific and precocious marker of MPS in general. Vertebral bodies appear flattened and beaked, potentially leading to later complications such as spinal nerve entrapment, acute spinal injury, and atlanto-occipital instability; thus, special care must be taken to prevent dislocation of the atlanto-axial joint during general anaesthesia and surgery. After 1 year of age, joint contractures and genu valgum represent other frequent signs of joint impairment and progressive skeletal dysplasia, which are easily detectable at earlier than 2 years of age in all infants affected by MPS. All these skeletal manifestations together, typical of MPS, are named dysostosis multiplex. Thickening of the ribs can be seen on radiographs (Fig. 3a), the long bones are short with wide shafts, and the knees are prone to valgus and varus deformities. Phalangeal dysostosis and synovial thickening lead to a characteristic claw hand deformity (Fig. 4), which is another typical feature that may raise suspicion of the disease. Hip dysplasia is frequently the reason why these children may be first seen by orthopaedic specialists. Typically, the pelvis is poorly formed, the femoral heads are small, and coxa valga is common, with progressive and debilitating hip deformity (Fig. 3b) [14, 16].

There is a notable difference between MPS I and MPS II; in MPS I, skeletal growth decelerates by 3 years of age [14], while MPS II patients show overgrowth in the first 5–6 years of age, slowing their growth thereafter [4].

Coarsening of the facial features (broad nose with flared nostrils, prominent supraorbital ridges, large rounded cheeks, thick lips, enlarged protruding tongue) caused by storage of GAGs in the soft tissues of the orofacial region and facial bones becomes apparent within the first 2 years, at a median age of 1.1 years for Hurler disease [12] and between 18 months and 4 years in the severe form of Hunter disease [16], although subtle phenotypic changes can be detected earlier (as early as the second half of the first year) by paediatricians with specific experience (Fig. 1). Macrocephaly becomes progressively more evident, and scaphocefaly is common (Fig. 5), but microcephaly is also possible in Hurler patients [17]. Facial and body hirsutism are always evident by the age of 24 months [14]. The skin may be thickened and inelastic. Notably, some MPS II patients develop a distinctive skin lesion, which is described as ivory-white papules (pebble-like) on the upper back and sides of the upper arms, pathognomonic of Hunter syndrome [18].

Cardiomyopathy and progressive thickening and stiffening of the valve leaflets in MPS I patients can lead to mitral and, less frequently, aortic regurgitation [14]. A murmur may be detected in the first months of life and has sometimes be reported as the first sign of the disease [19]. Cardiac involvement is also present in almost all patients with MPS II, but this starts at around 5 years of age [10]. Valve disease may lead to right and left ventricular hypertrophy and heart failure.

Protuberance of the abdomen caused by progressive hepatosplenomegaly is easily evident after 6 months of age, although storage of GAGs in the liver and spleen does not lead to organ dysfunction [14].

Corneal clouding occurs in almost all individuals with MPS I at earlier than 15 months of age [12], potentially causing severe visual impairment. On the contrary, corneal clouding is not a typical feature of Hunter syndrome [16], but slit-lamp examination may reveal discrete corneal lesions that do not affect vision. Retinal dysfunction may be detected, while glaucoma is not a common finding.

Early psychomotor development is generally detected as normal, but in MPS I developmental delay is usually obvious by the age of 18 months, with a progressive mental decline leading to a severe intellectual disability. Language skills are very limited in these children. A global delay of developmental milestones in MPS II becomes evident from 2 years onward [13]. However, the leading neurological feature of severe Hunter disease is represented by marked behavioural disturbances, such as hyperactivity, obstinacy, and aggressiveness, that are typically not observed in those with the attenuated phenotype [8].

Mucopolysaccharidosis type VII (MPS VII; OMIM #253220), also known as Sly syndrome, is an ultra-rare MPS characterized by deficient activity of β-glucuronidase, with lysosomal storage of chondroitin sulphate (CS), DS, and HS, leading to cellular and organ dysfunction. The first patient was described by Sly et al. in 1973 [19] and a total of 145 patients have been reported in the literature up to now.

The clinical presentation and disease progression of MPS VII spans a wide severity spectrum, from early, severe, multisystem manifestations and death in the very first months, to a milder phenotype with later onset, normal or near-normal intelligence, and longer survival [20].

Phenotype characteristics of patients with MPS VII resemble those of MPS I and MPS II (short stature, skeletal dysplasia, macrocephaly, gingival hypertrophy, recurrent ear infections, hepatosplenomegaly, hernias, cardiac involvement, decreased pulmonary function, and cognitive impairment). An unexpectedly high proportion of described patients (41%) have a history of non-immune neonatal hydrops fetalis (NIHF) [21]. Despite the prenatal onset of the disease in these patients, 13 out of 23 survived infancy with a mild to intermediate course into their late teens. Thus, the presence of NIHF does not, by itself, predict the possible severity of the clinical course if the patient survives infancy. Another early sign is macrocrania, while heart valve involvement and repeated infections of the upper and lower respiratory tract appear in the first 2 years of life. Moderate mental retardation and progressive hearing loss with speech impairment are also evident with time.

In summary, the severe forms of MPS I have a very early multisystem onset (within 6 months of life); MPS II is similar but with a later onset, and MPS VII has a prenatal onset with frequent NIHF and early severe involvement of the upper and lower respiratory tract.

#### MPS with mostly neurological and cognitive involvement

**Fig. 7:**
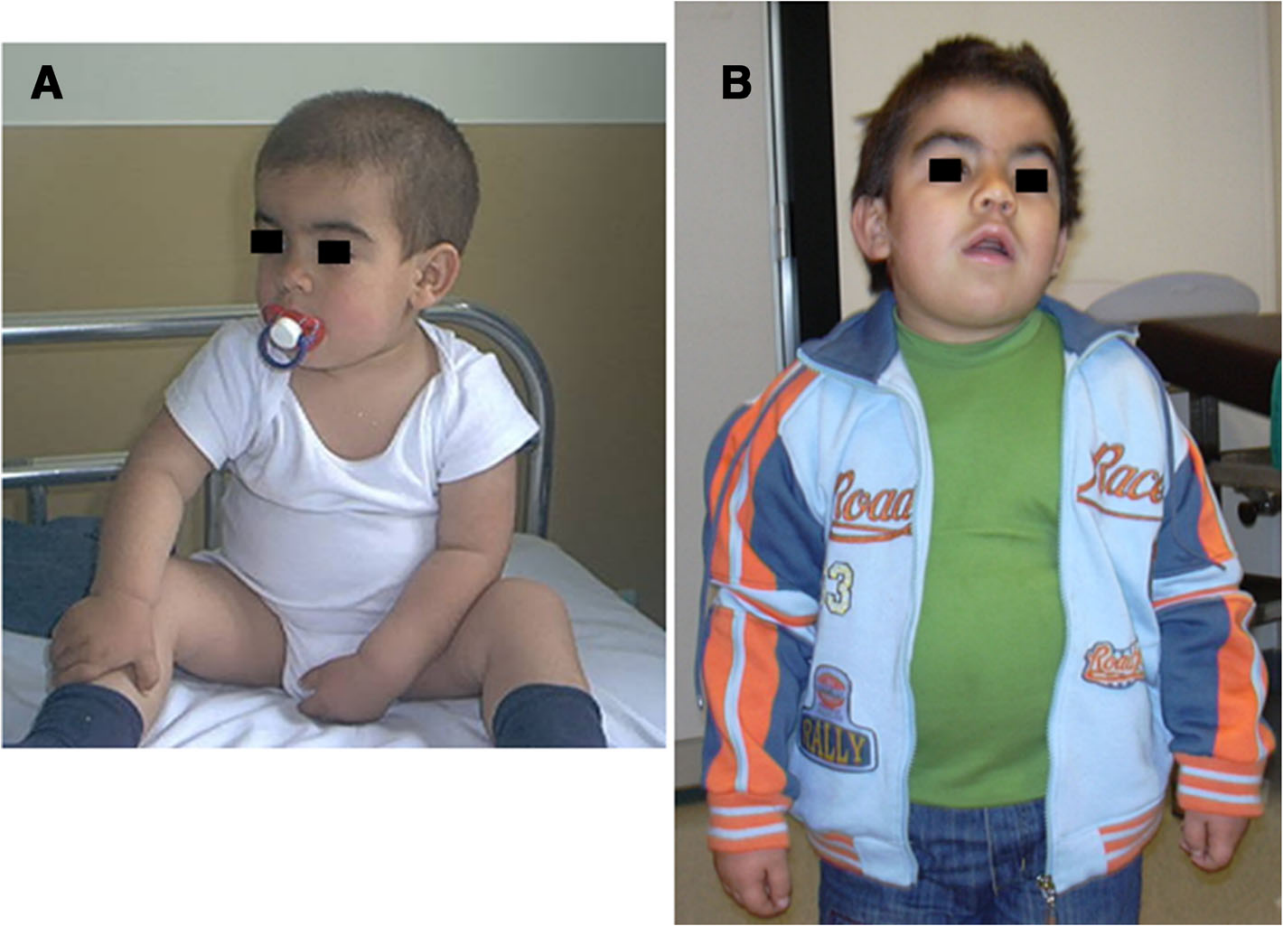
MPS IIIA. The same patient at **a** 2.5 and **b** 5 years old. Note the different face expression, showing progression of the neurological impairment

Mucopolysaccharidoses type III (MPS III, also known as Sanfilippo syndrome; Fig. 7) has a prevalent neurological presentation. MPS III is a lysosomal storage disorder caused by deficiency of one of the four enzymes involved in the catabolism of HS. Four different subtypes are known—MPS III type A (OMIM #252900), type B (OMIM #252920), type C (OMIM #252930), and type D (OMIM #252940)—each due to a different enzyme deficiency: type A, sulfamidase/SGSH gene; type B, alfa-*N*-acetylglucosaminidase/NAGLU gene; type C, alfa-glucosaminide *N*-acetyltransferase/HGSNAT gene; type D, *N*-acetilglucosamina-6-solfato sulfatasi/GNS gene). All four types (A to D) have autosomal recessive inheritance. MPS III is the most frequent of the MPS with an estimated prevalence between 0.3 and 4.1 cases for every 100,000 newborns, depending on the subtype and race [6].

Patients with Sanfilippo syndrome show progressive cognitive impairment in the second and third years of life. In contrast to the majority of MPS, somatic features are relatively less evident, and the CNS involvement is conspicuous. Speech development delay is usually the first sign noticed by the parents [22] but the main concerns can be behavioural problems (hyperactivity, anxious and aggressive behaviour, sleep disturbances), followed by progressive mental decline [22]. These symptoms can cause misdiagnosis as behavioural disturbances, attention deficit hyperactivity disorder (ADHD), and autism spectrum disorders [23]. Epilepsy may also be present.

Sleep disorders, the incidence of which is reported up to 80–90% [24], are a common feature. They consist of difficulties in falling asleep and frequent nocturnal awakening, with complete reversal of the day–night rhythm in some patients.

All these manifestations are very difficult to manage and have an enormous psychological impact on the quality of life for the whole family.

In general, although the phenotype can be very similar in the four subtypes, the clinical course in Sanfilippo B and C seems to be characterized by a less severe phenotype [25, 26].

Non-neurological symptoms are usually less pronounced in MPS III than in the other MPS. Recurrent ear, nose, and throat infections are observed at a younger age. Recurrent otitis, middle ear ossicle defects, and abnormalities in the inner ear can cause deafness. An excess of thick secretions and anatomical changes can produce obstruction of the airways. In the first years of life, diarrhoea is frequently reported, whereas constipation is more common in older patients.

Physical examination can show coarse facial features, although these are less pronounced. Patients have macrocephaly, broad eyebrows with medial flaring and synophrys, the hair is usually dry and coarse, and hypertrichosis is common. In younger patients, mild hepatomegaly and umbilical and inguinal hernias are frequently observed. Rare, and often mild, contractures are mostly found in the elbows. Growth is affected in approximately half of patients older than 12 years [25].

In summary, MPS III or Sanfilippo disease has a prominent neurological involvement with very mild somatic signs. Toddlers aged 2–3 years old developing hyperactivity, ADHD, and aggressive behaviour may be suspected of having MPS III.

#### MPS with prevalent somatic involvement

MPS IV and MPS VI present with only somatic involvement. These are progressive conditions that spare intellectual ability and mainly affect the skeleton (MPS IVA), or all the organs/systems of the body (MPS VI). Inheritance is autosomal recessive. The rate at which symptoms worsen varies among affected individuals.

**Fig. 8:**
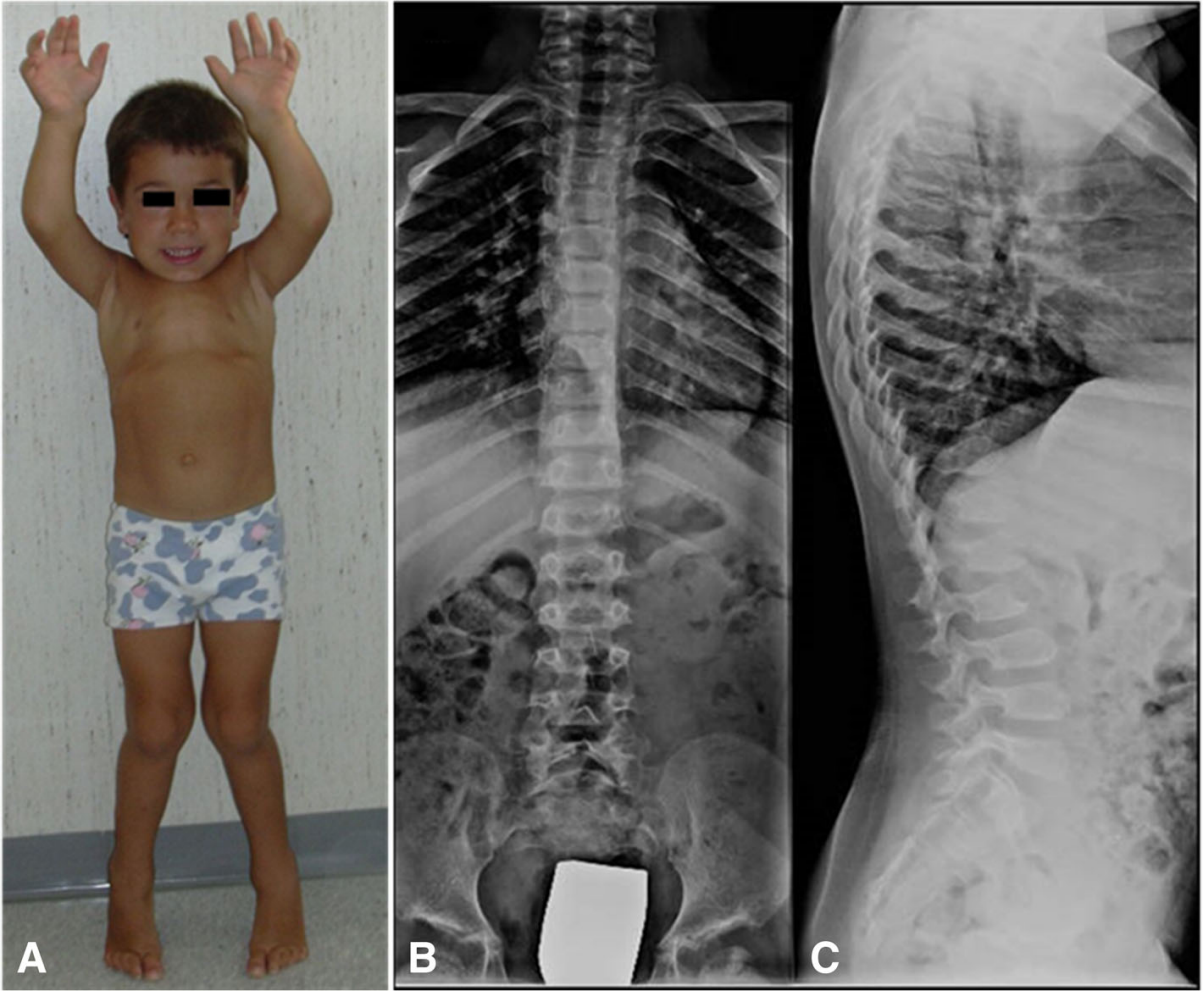
MPS IVA. **a** 4 year-old patient with **b** antero-posterior and **c** lateral x-rays showing “paddle-shaped” ribs and flattened and rounded vertebrae with a typical “anterior beaking” aspect

Mucopolysaccharidosis IV (MPS IV, also known as Morquio syndrome; Fig. 8) presents as two genetically distinct disorders, each with a different enzyme deficiency: MPS IVA (Morquio A syndrome; OMIM #253000) due to deficiency of *N*-acetylgalactosamine-6-sulfatase (GALNS gene), resulting in accumulation of keratan-sulphate (KS) and CS [26, 27]; and MPS IVB (Morquio B syndrome; OMIM #253010) due to deficiency of beta-galactosidase activity leading to urine KS and oligosaccharide excretion as in GM1 patients. MPS IVB is indeed allelic to the various forms of GM1-gangliosidosis, but lacks the psychomotor deterioration seen in GM1 gangliosidosis [28, 29].

Patients with severe MPS IVA with no detectable enzyme activity reveal their first symptoms usually during the first year of life, although they are rarely diagnosed before 4–5 years of age. Information on the natural history of Morquio A patients is mainly derived from two wide-ranging collections of data [28]. Reduced growth rate begins at approximately 18 months of age and growth will stop at approximately 7 or 8 years of age. In contrast to the other MPS, Morquio A joints are usually lax and very flexible (hypermobile), but some joints (usually the hips and shoulders) may have a restricted range of motion. Patients with such skeletal involvement may receive a diagnosis of, or undergo evaluation for, spondyloepiphyseal dysplasia, pseudoachondroplasia, multiple epiphyseal dysplasia, or bilateral Legg-Calvé-Perthes disease [30].

A constant feature is odontoid hypoplasia; thus, dislocation of the atlanto-occipital joint can occur causing compression and damage to the bulbar and spinal cord, resulting in paralysis or even death if cervical stabilisation is not performed early.

MPS IVB patients have a very similar clinical manifestation but with a less prominent short stature. They have mildly coarse facial features, hearing loss, corneal opacities, valvular heart disease, and frequent upper respiratory tract infections. They may also present with inguinal hernia and mild hepatomegaly. Skeletal dysplastic features include platyspondyly, odontoid hypoplasia with possible cervical subluxation, kypho-scoliosis, coxa valga, and constricted iliac wings [28].

**Fig. 9:**
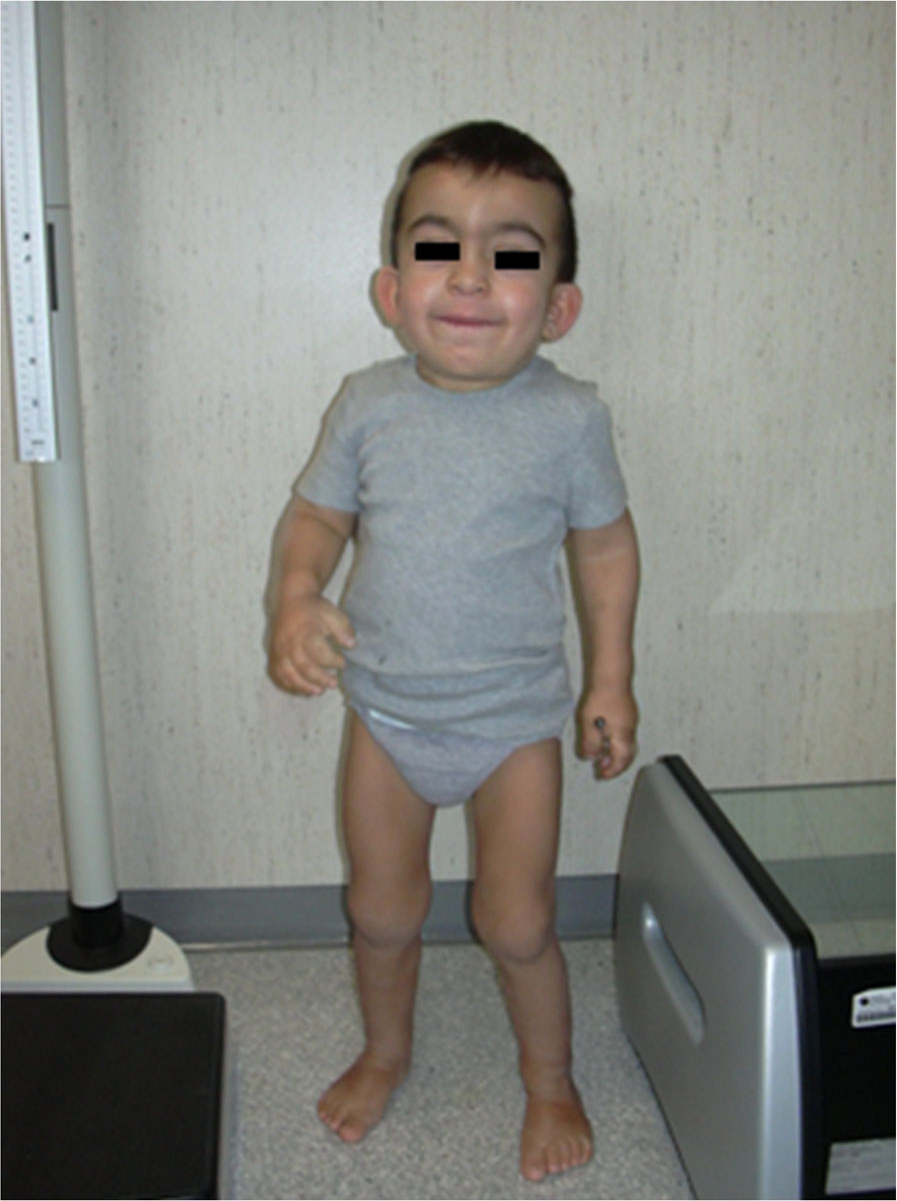
MPS VI. A 2-year-old boy with evident coarse face and skeletal dysostosis

Mucopolysaccharidosis VI (MPS VI, also known as Maroteaux-Lamy syndrome; OMIM #253200; Fig. 9) is due to pathogenic mutations in the arylsulfatase B (*ARSB*) gene located on chromosome 5. As a consequence, reduced or absent activity of the enzyme arylsulfatase B (ASB) (*N-*acetylgalactosamine 4-sulfatase) impairs degradation of DS determining its cellular accumulation [31].

Patients with no detectable enzyme activity have the severe form of MPS VI and exhibit symptoms in early childhood. Their somatic phenotype is similar to Hurler syndrome. In most cases, by 2 or 3 years of age, severe and progressive skeletal damage, named dysostosis multiplex, becomes evident. This skeletal dysplasia includes hip dysplasia with dysplastic femoral head, abnormal vertebral bodies, irregular clavicles, hypoplastic distal ulna and radius, and dysplastic and short metacarpal bones; carpal and tarsal bones are hypoplastic and have an irregular profile. Growth velocity often slows after the first year of life, with a final height generally lower than 120 cm. As in other MPS, an early sign is coarse facial features. In addition, patients have a typical protruding abdomen, hepatomegaly, umbilical and/or inguinal hernia, and heart involvement in early infancy. Although no primary intellectual disability is present, hydrocephalus can be seen in some patients with consequent intracranial hypertension and papilledema. Pectus carinatum, stiff and contracted joints, scoliosis and kyphosis, and cervical spinal cord compression worsen with time [32].

In summary, MPS IV and VI do not show CNS involvement. MPS IV is a unique MPS in showing joint laxity, while severe MPS VI onset is similar to that observed in MPS I.

## Conclusions

Symptoms and signs suggestive of MPS can lead to misdiagnosis or be underestimated if not considered as a continuum. Although many of these features have other possible causes, they should promptly arouse suspicion of MPS in any infant or child presenting with one or more of them. Referring these patients to a metabolic centre will allow the recognition of these disorders at a very early stage.

We want to stress the need for increased awareness of MPS among paediatricians and other specialists to shorten the still existing diagnostic delay [33]. While many attempts have been made to set affordable newborn screenings for this group of disorders, a timely diagnosis is mandatory in view of a possible early treatment when available. Many pilot studies on newborn screening for MPS I are at present ongoing with the aim of detecting the disease in a pre-symptomatic phase. This will bring new pros and cons which should be carefully evaluated over the upcoming years [34] (for a deeper discussion of newborn screening in MPS, see Donati et al. in this Supplement [35]).

## Abbreviations

ADHD: Attention deficit hyperactivity disorder
ASB: Arylsulfatase B
CNS: Central nervous system
CS: Chondroitin sulphate
DS: Dermatan sulphate
GAG: Glycosaminoglycan
HS: Heparan sulphate
KS: Keratan sulphate
MPS: Mucopolysaccharidosi(e)s
NIHF: Non-immune hydrops fetalis
